# Safety and Efficacy of Fecal Microbiota Transplantation in Treatment of Inflammatory Bowel Disease in the Pediatric Population: A Systematic Review and Meta-Analysis

**DOI:** 10.3390/microorganisms11051272

**Published:** 2023-05-12

**Authors:** Mark Hsu, Kyaw Min Tun, Kavita Batra, Lubaba Haque, Tahne Vongsavath, Annie S. Hong

**Affiliations:** 1 Department of Internal Medicine, Kirk Kerkorian School of Medicine at UNLV, University of Nevada, Las Vegas, NV 89102, USA; 2 Department of Medical Education, Kirk Kerkorian School of Medicine at UNLV, University of Nevada, Las Vegas, NV 89102, USA; 3 Office of Research, Kirk Kerkorian School of Medicine at UNLV, University of Nevada, Las Vegas, NV 89102, USA; 4 Division of Gastroenterology and Hepatology, Department of Internal Medicine, Kirk Kerkorian School of Medicine at UNLV, University of Nevada, Las Vegas, NV 89102, USA

**Keywords:** fecal matter transplantation, inflammatory bowel disease, Crohn’s disease, ulcerative colitis, pediatric

## Abstract

**Background and Aims:** Fecal microbiota transplantation (FMT) has been increasingly studied in the inflammatory bowel disease (IBD) population. However, most studies have focused on the adult population, and the safety and efficacy of FMT in a pediatric population is less well understood. This systematic review and meta-analysis investigates the safety and efficacy of FMT in a pediatric IBD population. **Methods:** A comprehensive literature search of publications published prior to 30 June 2022 was undertaken. Safety data, IBD-related outcomes, and microbiome analysis were obtained from these studies when accessible. Individual estimates of each study were pooled, and sensitivity analysis was conducted. **Results:** Eleven studies satisfied our eligibility criteria. The calculated pooled rate of adverse events was 29% (95% confidence interval [CI]: 15.0%, 44.0%; *p* < 0.001; I2 = 89.0%, Q = 94.53), and the calculated pooled rate of serious adverse events was 10% (95% confidence interval [CI]: 6.0%, 14.0%; *p* = 0.28; I2 = 18.0%, Q = 9.79). One month after FMT, clinical response was achieved in 20/34 (58.8%) pediatric IBD patients, clinical remission was achieved in 22/34 (64.7%), and both clinical response and remission were achieved in 15/34 (44.1%) pediatric IBD patients. **Conclusions:** FMT can be a safe and effective treatment in the pediatric IBD population and may demonstrate improved safety and efficacy in the pediatric population compared to the adult population. However, our results are limited by a lack of established protocol as well as long-term follow-up for FMT in a pediatric IBD population.

## 1. Introduction

Inflammatory bowel disease (IBD), typically subdivided into Crohn’s disease (CD) and ulcerative colitis (UC), is a chronic inflammatory condition of the gastrointestinal tract that arises from a complex interplay of genetic, environmental, and microbial factors resulting in dysregulated immune function [1]. Nearly 25% of IBD patients present under 20 years of age, with the incidence in this age group rising, making IBD an important area of focus in the pediatric population [2]. Due to the complex pathophysiology of IBD, optimal disease control is difficult despite a multitude of therapies already on the market [3]. Many existing therapies have notable limitations, such as lack of efficacy for certain patient populations, reduction of efficacy over time, high cost, and significant side effects [3]. These factors are further compounded in pediatric populations who may require therapy for a longer duration of time compared to those diagnosed later in life.

One therapy currently under investigation for IBD treatment is fecal microbiota transplantation (FMT), which involves the transfer of fecal matter from a donor to a recipient with the goal of changing the recipient’s gut microbiome composition. The donor stool is then introduced into a recipient’s gastrointestinal tract by means of endoscopy or capsule [4]. The gut microbiome population within a human host has been estimated to be greater than 100 trillion—over 10 times the number of human cells—and specific patterns in alterations of the gut microbiome have been identified in IBD patients [4,5,6]. FMT has been most extensively studied in Clostridium difficile infection (CDI), but the use of FMT in IBD has received increased attention particularly over the last decade [7]. Systematic reviews and meta-analyses in the last few years have resulted in common findings of potential benefit of FMT in clinical remission of UC and unclear impact in CD, with significant limitations noted due to small sample size and poor quality of studies [7,8,9].

The majority of these FMT studies in IBD populations have not distinguished between pediatric and adult populations, making it difficult to ascertain efficacy and safety of FMT specifically within the pediatric IBD population. Understanding the role of FMT in pediatric IBD is particularly important given that the recent literature has suggested a delicate and dynamic microbiome composition throughout childhood and adolescence that is especially susceptible to environmental factors [10]. This raises the possibility of microbial manipulation via FMT being more impactful in a pediatric population relative to adults. Unfortunately, a recent review of FMT in all ages of IBD acknowledged that most of the literature on FMT in a pediatric IBD population has been limited to case reports and case series [11]. Furthermore, much of the literature pertaining to FMT in pediatric IBD is in the context of CDI [11,12]. This limitation of sample size was also noted in the only systematic review of FMT in pediatric IBD to our knowledge [13]. 

Since these aforementioned reviews, FMT has received significant attention among researchers, with numerous studies published in the interim contributing additional data points for the safety and efficacy of FMT in pediatric IBD. Therefore, we aim to conduct an updated systematic review to evaluate the safety and efficacy of FMT in pediatric IBD.

## 2. Materials and Methods

### 2.1. Search Strategy

We performed a comprehensive literature search across five databases (PubMed/Medline, Embase, CINAHL, Cochrane, and Web of Science) using variations of the keywords “fecal microbiota transplant” and “pediatric” to identify original studies published from inception through to 30 June 2022. Results were limited to human studies published in English.

Prior to screening the studies for eligibility to be included in the systematic review and meta-analysis, our review was reported according to the preferred reporting items for systematic review (PRISMA) guidelines, and it was registered on PROSPERO (registration number CRD42022343342).

### 2.2. Eligibility Criteria

We included studies that met the following inclusion criteria: (1) FMT in a patient diagnosed with IBD; (2) pediatric population; (3) reporting of patient data and outcomes after first fecal infusion; (4) patients of any sex; (5) minimum follow-up of 2 weeks; and (6) at least moderate quality of evidence. Although we acknowledge the American Academy of Pediatrics (AAP) eliminating an upper age limit for the betterment of clinical care in their most recent guidelines, we required an age cut-off for the purposes of our study and defined a pediatric population as 21 years or younger in accordance with the AAP’s most recent definition of a pediatric population prior to eliminating an upper age limit [14]. Additionally, characteristics and data of IBD patients needed to be clearly delineated to include a study that did not otherwise focus purely on IBD.

We excluded studies that met the following exclusion criteria: (1) case reports or case series with less than 5 patients to minimize bias based on a prior concept analysis [15]; (2) published abstracts, letters to editor, and commentaries which do not require detailed patient data or an extensive review process; (3) studies without patient data; (4) non-English studies; and (5) animal studies.

### 2.3. Quality Assessment

Revised Cochrane risk-of-bias tool (RoB 2) was used to evaluate the methodological quality of randomized controlled trials (RCT). RoB 2 is a revised version of the original Cochrane risk-of-bias tool that has been widely used in systematic reviews. The tool consists of five domains: randomization process, derivations from intended interventions, missing outcome data, measurement of the outcome, and selection of the reported result. The overall risk of bias for each RCT is determined as high, low, or some concern based on the individual elements in the 5 domains [16].

The Newcastle–Ottawa Scale (NOS) was used to evaluate the methodological quality in case-control and cohort studies. Risk of bias regarding the selection of subjects, comparability of subjects, and assessment of the exposure and outcome was graded by using a star system corresponding to nine items. A study was categorized as low risk of bias if a total of 8 to 9 stars were allocated, medium risk of bias if 6 to 7 stars were allocated, and high risk of bias if the study was given ≤5 stars [17].

A series of quality assessment tools developed by the US National Heart Lung and Blood Institute (NHLBI) of National Institutes of Health (NIH) (https://www.nhlbi.nih.gov/health-topics/study-quality-assessment-tools) (accessed on 1 August 2022) was used to determine methodological quality and risk of bias for case series. Similarly to NOS, a set of question items with yes/no answers were used, with a “Yes” counting as a score of 1 and a “No” as a score of 0. In the tool used for case series, there were a total of 9 questions. A score of 7–9 corresponds to good quality, while scores of 4–6 and 1–3 indicate moderate and poor quality, respectively [18].

In the final selection stage, only studies with at least a moderate level of evidence were included. Quality appraisal was performed by at least two of the following authors: M.H., L.H., and T.V. If there was any disagreement, a senior reviewer (K.B.) evaluated the article and achieved consensus through discussion.

### 2.4. Study Selection and Data Extraction

**Figure 1:**
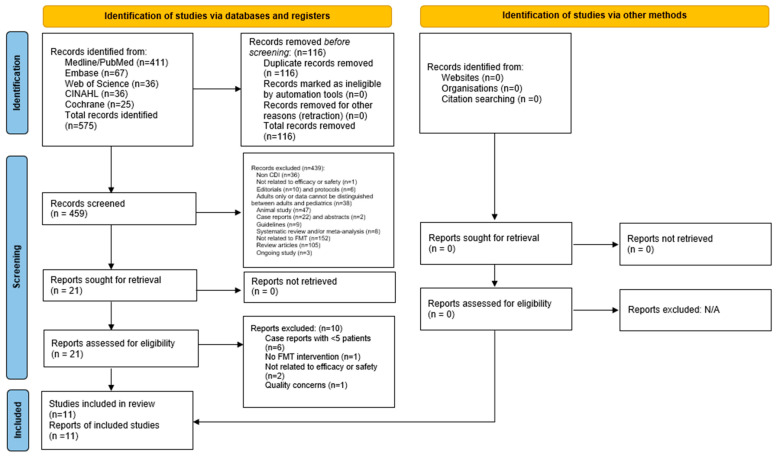
Preferred reporting items for systematic review and meta-analysis (PRISMA).

A total of 575 articles were retrieved in the initial search. Two authors (K.T. and M.H.) independently reviewed these titles and abstracts, after which 21 articles were deemed relevant with patient data. Full texts were then reviewed by at least two of the following authors: M.H, T.L., and T.V. Following this, the 11 remaining studies fulfilled the complete eligibility criteria. In cases of disagreement, a senior reviewer (A.S.H.) arbitrated the final decision for inclusion. The study selection process by Preferred Reporting Items for Systematic Reviews and Meta-Analyses (PRISMA) statement is detailed in Figure 1. A summary of included studies is shown in Table 1. An IRB review was not required as all data were extracted from the published literature and no patient intervention was directly performed. The data underlying this article will be shared on reasonable request to the corresponding author.

### 2.5. Study Outcomes

The primary outcome of this quantitative analysis was the safety of FMT in the treatment of pediatric IBD patients. Safety was assessed through the event rate of adverse events (AEs) and serious adverse events (SAEs) of FMT treatment. An SAE was defined as hospitalization for any reason and death. The event rate was calculated by dividing the number of patients experiencing adverse events or serious adverse events by the total sample size of the individual studies.

The secondary outcome for our study was the efficacy of FMT with respect to disease severity in pediatric IBD patients. Efficacy was categorized as clinical response and clinical remission. Clinical response was defined as greater than or equal to a 20-point decrease in the Pediatric Ulcerative Colitis Activity Index (PUCAI) in UC patients, or greater than a 12.5-point decrease in the Pediatric Crohn’s Disease Activity Index (PCDAI) in CD patients. Clinical remission was defined as PUCAI or PCDAI under 10. Although source authors may have applied different markers of clinical response and/or remission, our definitions are consistent with the literature for PUCAI and PCDAI [19,20,21,22].

In addition to efficacy and safety, we examined the effect of FMT on the gut microbiome of pediatric IBD patients.

### 2.6. Data Analysis

Individual estimates of each study were pooled to compute the summary estimates of FMT’s safety. A weighted summary statistic was calculated if many zero values occurred (e.g., adverse events) to prevent positive bias. A random effects model was fitted to account for methodological differences among included studies for generating summary estimates [23]. The strength of evidence of heterogeneity across studies was determined by Cochran’s Q and I2 statistics [24,25,26]. Values of under 30%, 30–60%, 61–75%, and over 75% were categorized as having low, moderate, substantial, and considerable heterogeneity, respectively [27]. Sensitivity analysis was conducted to determine the validity of the estimated summary effect size. Publication bias was assessed by visually inspecting the funnel plot and doi plot [28,29]. In addition, the Luis Furuya-Kanamori (LFK) index was used as a quantitative method to assess the asymmetry of the study effects or publication bias as it has been noted in the literature that the LFK index has higher sensitivity than the Egger regression statistics, particularly in a meta-analysis with a small number of studies [28]. All meta-analyses were performed using MetaXL software (v. 5.3; EpiGear International, Sunrise Beach, Queensland, Australia). The 95% Clopper–Pearson exact confidence intervals were calculated using the R package [30,31]. In our meta-analysis of safety, a leave-one-out analysis was conducted to assess if any study involved in the main analysis had a dominant effect. As shown in Table 1, upon removal of each study one by one, no significant impact on the summary statistics of the main outcome or heterogeneity was found. 

## 3. Results

As shown in Table 1, a total of 11 studies were included in our study. In total, there were 352 pediatric IBD patients who underwent FMT in these studies. Table 2 summarizes the FMT protocol for each study.

**Table 1:** Summary of included studies including patient characteristics.

Author/Year	Study Design	Quality Assessment Tool	Quality Score	Number of IBD Patients Who Received FMT	Age Range (Years)	Gender	Comorbidities	Follow-Up Period (Weeks)
Nicholson et al., 2022 [32]	Retrospective	NOS	8	148	≤21	*	C. diff infection	>12
Nicholson et al., 2020 [33]	Retrospective	NOS	8	130	≤21	*	C. diff infection	>8
Cho et al., 2019 [34]	Case series	NIH	7	8	9–18	5 male 3 female	C. diff infection	6–94
Fareed et al., 2018 [35]	Prospective	NOS	8	5	7–16	2 male 3 female	C. diff infection	Up to 36
Karolewska-Bochenek et al., 2021 [36]	Case series	NOS	7	8	1.5–16.5	4 male 4 female	CMV colitis	2, 6
Karolewska-Bochenek et al., 2018 [37]	Case series	NOS	6	10	10–17	3 male 7 female	None	2.5–5
Goyal et al., 2018 [38]	Case series	NOS	9	21	8–21	12 male 9 female	None	1, 4, 24
Hourigan et al., 2015 [39]	Case series	NOS	8	5	10–17	NR	C. diff infection	2–24
Suskind et al., 2015 [40]	Case series	NOS	7	9	12–19	5 male 4 female	None	2, 6, 12
Kunde et al., 2013 [41]	Case series	NOS	6	10	7–20	6 male 4 female	None	6
Shimizu et al., 2019 [42]	Case series	NOS	5	8	2–9	NR	None	Up to 52

* In both Nicholson studies, gender was not specified within the IBD population.

**Table 2:** Details on FMT administration.

Author/Year	Administration Method and Number of Patients	Number of Administrations	Volume of Instilled FMT per Dose (g/mL)	Donor Details
Nicholson et al., 2022 [32]	Colonoscopy, sigmoidoscopy, EGD, NG, ND, NJ, capsule	1	NR	Patient-selected, commercial and local stool banks
Nicholson et al., 2020 [33]	Colonoscopy, NG, NJ, capsule, enema, sigmoidoscopy	1	240 mL	Patient-selected, commercial and local stool banks
Cho et al., 2019 [34]	Colonoscopy	1–2	NR	Commercial stool bank, screened family member
Fareed et al., 2018 [35]	Colonoscopy, NJ	1	NJ: 60 mL, colonoscopy up to 240 mL	Commercial stool bank
Karolewska-Bochenek et al., 2021 [36]	NG	1	30–60 g in 50–100 mL	Screened and unrelated donors
Karolewska-Bochenek et al., 2018 [37]	ND, gastroscopy	5	50 g in 50 mL	Screened and unrelated donors
Goyal et al., 2018 [38]	Colonoscopy	1	150 g in 250–300 mL	Healthy family members, first-degree relatives, or trust friends
Hourigan et al., 2015 [39]	Colonoscopy	1	Up to 100 g in 400 mL	Related donors aged 24–56
Suskind et al., 2015 [40]	NG	1	30 g in 100–200 mL	Related (parents)
Kunde et al., 2013 [41]	Enema	20	70–113 g in 250 mL	Screened family members and close friends aged >18
Shimizu et al., 2019 [42]	Colonoscopy, enema	3–5	NR	Related (parents or unspecified family member)

### 3.1. Safety

**Figure 2:**
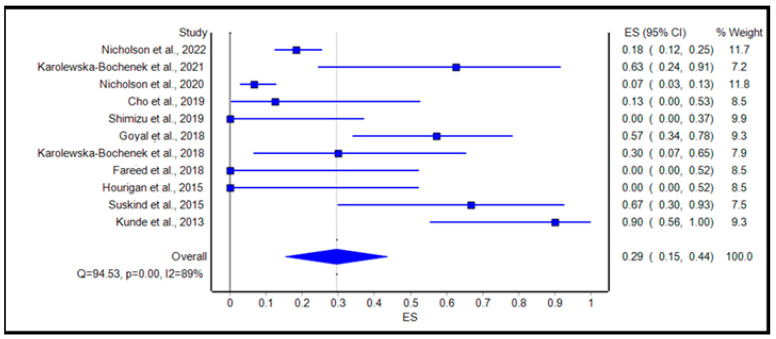
Forest plot displaying the pooled rate of adverse events of FMT among IBD patients. References: Nicholson et al., 2022 [32]; Karolewska-Bochenek et al., 2021 [36]; Nicholson et al., 2020 [33]; Cho et al., 2019 [34]; Shimizu et al., 2019 [42]; Karolewska-Bochenek et al., 2018 [37]; Goyal et al., 2018 [38]; Fareed et al., 2018 [35]; Hourigan et al., 2015 [39]; Kunde et al., 2013 [41]; Suskind et al., 2015 [40].

**Figure 3:**
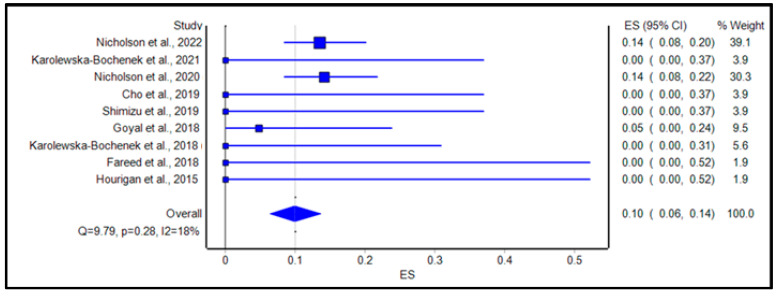
Forest plot displaying the pooled rate of serious adverse events of FMT among IBD patients. References: Nicholson et al., 2022 [32]; Karolewska-Bochenek et al., 2021 [36]; Nicholson et al., 2020 [33]; Cho et al., 2019 [34]; Shimizu et al., 2019 [42]; Karolewska-Bochenek et al., 2018 [37]; Goyal et al., 2018 [38]; Fareed et al., 2018 [35]; Hourigan et al., 2015 [39].

Table 3 reviews the AEs and SAEs in our studies. The calculated pooled rate of AEs was 29% (95% confidence interval [CI]: 15.0%, 44.0%; *p* < 0.001; I2 = 89.0%, Q = 94.53), as shown in Figure 2. The calculated pooled rate of SAEs was 10% (95% confidence interval [CI]: 6.0%, 14.0%; *p* = 0.28; I2 = 18.0%, Q = 9.79), as shown in Figure 3.

### 3.2. Efficacy

One month after FMT, clinical response was achieved in 20/34 (58.8%) pediatric IBD patients, and clinical remission was achieved in 22/34 (64.7%) pediatric IBD patients, as shown in Table 4. In total, 15/34 (44.1%) pediatric IBD patients demonstrated both clinical response and remission 1 month after FMT. To assess if any study involved in the main analysis had a dominant effect, a leave-one-out analysis was conducted. As shown in Table 5, upon removal of each study one by one, no significant impact on the summary statistics of the main outcome or heterogeneity was found.

In pediatric UC patients, 13/20 patients had clinical response to FMT, 10/20 patients had clinical remission, and 8/20 patients exhibited both clinical response and remission to FMT within 1 month; 5 patients had no benefit entirely. In one study, three patients were followed through 6 months, one of which continued to show both clinical response and remission, one of which had only clinical response, and one of which had no benefit.

In pediatric CD patients, 12/14 patients had clinical remission, and 7/14 patients had both clinical response and remission within 1 month; no patients demonstrated clinical response only, and 2 patients had no benefit entirely. In one study, nine patients were followed through 3 months, with three patients showing both clinical response and remission, three patients showing clinical remission only, and three patients showing no benefit. In another study, three patients were followed through 6 months, two of which showed clinical remission while one showed both clinical remission and response.

## 4. Discussion

To the best of our knowledge, our paper provides the first meta-analysis of the safety of FMT in pediatric IBD, the first systematic review of microbiome effects of FMT in pediatric IBD, and an update to the systematic review of safety and efficacy of FMT in pediatric IBD by Wang et al. in 2016 [13]. Since the systematic review by Wang et al., the number of pediatric IBD patients to undergo FMT has increased over tenfold. 

### 4.1. Safety

Our study demonstrated an overall pooled rate of 29% (95% CI: 15–44%, *p* < 0.001) for AEs and 10% (95% CI: 6–14%, *p* < 0.28) for SAEs. No deaths occurred in any FMT recipients, and all SAEs were related to hospitalizations. Importantly, while not all studies specified whether hospitalizations were related to infection, those that did reported no opportunistic infections related to the donor sample. Most AEs were mild, including abdominal pain, bloating, nausea, vomiting, and diarrhea. Many of these symptoms may be seen in IBD at baseline, and multiple studies acknowledged it was difficult to discern whether the symptoms were truly related to FMT or the underlying disease process. Additionally, it is important to consider that many patients pursue FMT only after more established treatments have failed. Several of our studies had inclusion criteria that specifically focused on refractory disease. A qualitative assessment among adults who underwent FMT for UC showed that many patients were motivated to pursue FMT as a last resort [43]. Furthermore, it is also difficult to distinguish between whether AEs were due to the donor sample itself or the route of FMT administration. Indeed, a questionnaire for participants of a FMT trial in pediatric UC patients revealed that most discomfort was related to the route of FMT administration and not complications that arose following FMT [44]. 

In comparison, a Cochrane systematic review by Imdad et al. from 2018 [8], which assessed IBD patients of all ages, found an AE rate of 78% and an SAE rate of 7%. Importantly, AEs and SAEs were reported in only two of their four studies, and none of their SAEs were attributable to death. In another meta-analysis of FMT safety in a UC population of all ages from 2022, SAEs occurred at a pooled rate of 10% [45]. Unfortunately, it is unclear if there was any mortality in this data set. Overall, these two studies were similar to ours in terms of SAEs. Our study had a significantly lower rate of AEs compared to Imdad et al., which may suggest better tolerability of FMT in the pediatric patients compared to adults. 

Although there were no clearly documented episodes of opportunistic infections in our data set, we agree with the United States Food and Drug Administration (FDA) recommendation to screen donor samples for multidrug resistant organisms (MDRO) given previous reports of death due to MDRO infections from donor samples [46]. 

### 4.2. Efficacy

Within 1 month of FMT in pediatric IBD patients, clinical response was achieved in 20/34 (58.8%) of patients, and clinical remission was achieved in 22/34 (64.7%). In comparison, a systematic review and meta-analysis of the efficacy of FMT in adult IBD patients by Tan et al. in 2022 showed a clinical response rate of 150/357 (42.0%) and a clinical remission rate of 168/455 (36.9%) [47].

This improved efficacy in the pediatric population may be explained by known patterns of gut microbiome development through adulthood. Although gut microbiome compositions are relatively stable, studies have suggested that it may take at least 3 years for the gut microbiome to mature, with recent studies suggesting further changes into teenage years [10,48]. During these years of maturation, the gut microbiome has been shown to be more susceptible to change compared to adulthood [49,50].

Importantly, there was a lack of consistency in donor sources, method of FMT administration, number of FMT administrations, or volume of FMT per administration among our reviewed studies, as shown in Table 3. Many of these factors may affect clinical response and remission rate. A previous systematic review and meta-analysis of all ages suggested improved chances of clinical remission with lower gastrointestinal tract administration of FMT as well as maintenance therapy with increased number of FMT administrations [7]. In our reviewed studies, there was an even mix of upper and lower gastrointestinal tract administrations, while most studies had only a single FMT administration. Additionally, appropriate age-matching between donors and recipients has been suggested to affect long-term efficacy of FMT [51]. None of the studies in our review used age-matched donors, with most donor sources consisting of older family members or public stool banks. Clarification on the impact of these variables can better optimize FMT protocols.

### 4.3. Microbiome Analysis

The gut microbiome, through its abilities in modulating the host immune system and metabolism as well as serving as a defense mechanism against pathogens, has been recognized as a critical component of IBD, but its clinical significance in the management and outcomes of IBD remain unclear [1,52]. As such, special attention has been given to FMT as a potential therapy for manipulating dysbiosis. Four studies in our review included a microbiome analysis [35,38,39,40].

Microbiome diversity within a single sample is referred to as α-diversity. Many studies have previously shown IBD patients to have reduced α-diversity, with increased α-diversity after FMT [7,53,54]. In all four of our studies that included a microbiome analysis, α-diversity was reduced in pediatric IBD patients but increased following FMT. Oftentimes, pre-FMT α-diversity was lower at baseline in IBD patients compared to non-IBD patients. The post-FMT increase in α-diversity was self-limited in these studies, and both Goyal et al. and Hourigan et al. noted a reversal back towards pre-FMT α-diversity after 6 months. Interestingly, Hourigan et al. noted that this reversal in α-diversity was noted only in IBD patients, whereas non-IBD patients maintained increased α-diversity after FMT through 6 months. Previously, a study estimated the sustained effect of a first FMT episode to be approximately 4 months [55]. This once again emphasizes the need for further investigation of the appropriate number of administrations in a FMT protocol. 

Microbiome diversity between different samples is referred to as β-diversity, with a narrower β-diversity indicating increased similarity between samples. In Goyal et al., β-diversity narrowed for 1 month after FMT but reverted back towards pre-FMT β-diversity at 6 months, whereas there was no change in β-diversity for IBD patients after FMT in Hourigan et al. Suskind et al. noted that the magnitude of response to FMT was related to the magnitude of divergence between donor and recipient microbiomes, with more differences between donor and recipient leading to more clinical response in the recipient. 

Specific trends in microbial composition have been associated with IBD. In both CD and UC, studies have noted a simultaneous increase in Enterobacteriaceae and decrease in Bacteroidales [56,57]. Goyal et al., Fareed et al., and Hourigan et al. all showed a decrease in Enterobacteriaceae after FMT, while Fareed et al. and Hourigan et al. both noted an increase in Bacteroidales after FMT. Notably, Enterobacteriaceae has been noted to have proinflammatory effects in the intestine, while Bacteroidales conveys anti-inflammatory effects [54].

Although some findings in our reviewed studies are in line with the prior literature, we also note some deviations. In Goyal et al., responders to FMT were noted to have a significantly higher abundance of Fusobacterium in their pre-FMT samples. In contrast, two previous studies found Fusobacterium abundance to be associated with lack of response [58,59]. Furthermore, Hourigan et al. showed that IBD patients have a relative increase in Fusobacterium after FMT. These inconsistencies raise the question of what is cause versus effect from FMT intervention and also emphasize the importance of considering confounding factors. Importantly, while FMT can certainly impact a recipient’s gut microbiome, many factors have been shown to manipulate the microbial colonization and proliferation, including diet, pH, and luminal transit time [52,54]. In combination, these factors serve as confounders that may impact the microbiome analysis of these studies. 

### 4.4. Limitations

Some limitations exist in our paper. First, as a systematic review and meta-analysis, the studies we analyzed were designed with varying protocols, resulting in clinical heterogeneity. Studies differed in how they reported AEs and how they defined efficacy of their FMT treatments. With AEs, studies deviated in clarifying if AEs were related to FMT or other clinical factors such as the underlying disease, distinguishing what defines an SAE, and reporting unique AEs encountered in the study as a whole versus reporting AEs of each individual patient that was affected. With efficacy, studies evaluated different lab markers of evaluation and clinical scores for disease activity and identified varying time frames for follow-up evaluation.

Second, there were no RCTs in our analysis to provide a controlled reference. Although there have been a handful of randomized control trials (RCT) in adult patients, there has only been one RCT on FMT in pediatric IBD to date [60]. Unfortunately, this RCT was unable to reach their recruitment target and also experienced significant dropout. Additionally, there was uncertainty in how this study defined clinical remission, as PUCAI scores of <10 and <15 were both mentioned. Due to these factors potentially affecting the quality of evidence, this RCT was excluded from our review. Several explanations were provided by the authors for their enrollment struggles, including patient hesitation after learning more about the trial protocol as well as patient withdrawal at the request of their primary clinical team due to lack of immediate signs of improvement from FMT. These beliefs are understandable given the novelty and uniqueness of FMT, particularly among the pediatric population. Multiple studies have examined hesitations from both patients and providers due to lack of knowledge, concerns over hygiene, and reservations concerning side effects, even in spite of documented patient satisfaction with FMT [61,62,63].

Third, as some of our studies evaluated FMT in pediatric IBD patients in the setting of other disease processes such as CDI and cytomegalovirus (CMV) colitis, the results of these studies may be confounded by these disease processes. As mentioned earlier, some studies did not clarify if the SAEs or AEs encountered were related to the FMT itself or the underlying disease process, and many of our recorded SAEs and AEs come from studies that involved other disease processes. Although we were unable to perform a subgroup analysis given the limited sample size, there is a possibility that SAEs and AEs directly related to FMT may be lower than what we report in our study, which lends further reassurance to the safety of FMT in the pediatric population. Additionally, as we only wanted to use studies without any comorbidities to reduce confounding variables, our sample size for efficacy analysis was greatly reduced.

**Figure 4:**
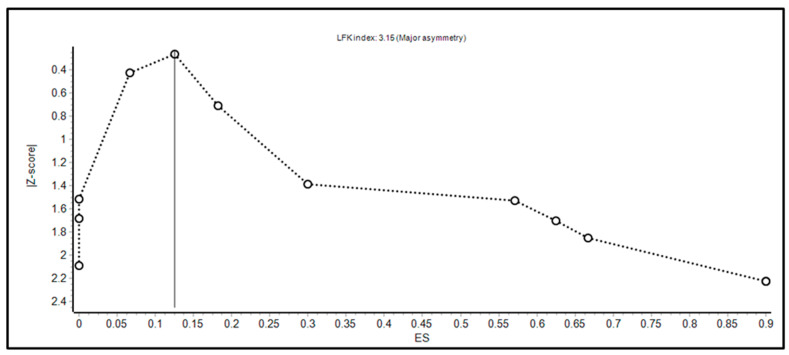
Doi plot for assessing the evidence of publication bias.

**Figure 5:**
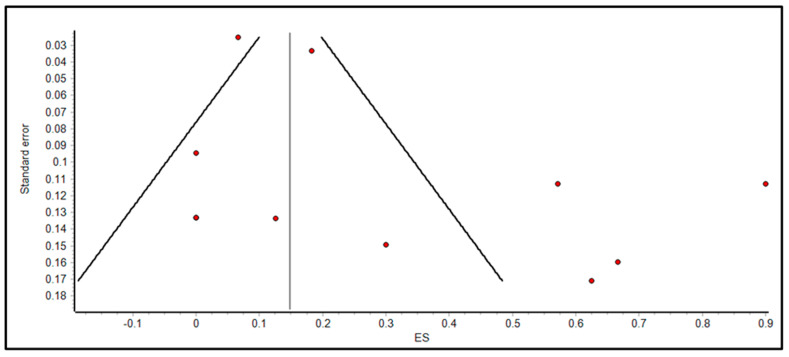
Funnel plot for assessing the evidence of publication bias.

Fourth, we were limited to defining efficacy of FMT in pediatric IBD to disease activity as defined by PUCAI and PCDAI, as these clinical scores were consistently reported by all studies that focused on FMT in IBD only. Otherwise, laboratory studies such as fecal calprotectin and C-reactive protein were inconsistently measured, and there were no reports on endoscopic healing. Fifth, the population pooled in the studies of our review and meta-analysis may not adequately represent most pediatric IBD patients, as FMT is typically only pursued in more severe cases where IBD is refractory to more established treatments. As we mentioned previously, this may affect how adverse effects are collected and reported, as well as their clinical significance. Sixth, as revealed by the doi plot in Figure 4 and the funnel plot in Figure 5, there was evidence of asymmetry, and potential publication bias was found.

## 5. Conclusions

Our review confirms the relative safety and efficacy of FMT in a pediatric IBD population compared to adults. The rate of AEs was lower in our pediatric population compared to the adult population, and the rate of SAEs was in line with prior reviews of all ages. Additionally, clinical response and clinical remission was achieved more in our pediatric population compared to published rates in adults. Our findings suggest some applicability to the pediatric population of certain trends noted in adult IBD microbiomes in response to FMT but also note some inconsistencies.

Although the results of our study are promising, the clinical applicability of these findings are still limited as FMT remains poorly regulated with no universally accepted protocols for preparation and administration of fecal samples. Indeed, our difficulty with achieving generalized conclusions despite an ever-increasing number of FMT data points over the past few years reflects the overwhelming need for clearer and more consistent protocols for FMT.

To achieve a better understanding of what an optimal FMT entails, future studies should aim to delineate differences in the efficacy of the method of administration, frequency of administrations, volume of fecal matter instilled per administration, and donor details, such as age, comorbidities, and gut microbial composition. Further clarity in the impact of these factors on FMT success will help to establish more optimal FMT protocols moving forward. Additionally, longer follow-up will inherently allow for a better understanding of the long-term safety and efficacy of FMT.

## Figures and Tables

**Table 3:** Adverse events and serious adverse events.

Author/Year	Number of FMT-Related SAEs	Number of Patients with SAEs	Number of FMT-Related AEs	Details of AEs
Nicholson et al., 2022 [32]	29	27	NR	NR
Nicholson et al., 2020 [33]	13	17	3	Diarrhea, abdominal pain, abdominal bloating
Cho et al., 2019 [34]	0	0	3	Fever, abdominal pain, influenza
Fareed et al., 2018 [35]	0	0	1	Abdominal pain
Karolewska-Bochenek, 2021 [36]	0	0	3	Abdominal pain, nausea, post-procedure vomiting
Karolewska-Bochenek, 2018 [37]	0	0	2	Nausea, post-procedure vomiting
Goyal et al., 2018 [38]	0	0	7	Abdominal pain, diarrhea, bloating and flatulence, vomiting, bloody stools, nausea, and fever
Hourigan et al., 2015 [39]	0	0	4	Abdominal pain, diarrhea, fecal incontinence, fecal urgency
Suskind et al., 2015 [40]	0	0	7	Abdominal pain, diarrhea, abdominal bloating, rhinorrhea, nasal congestion, sore throat, flatulence
Kunde et al., 2013 [41]	0	0	6	Abdominal cramping, abdominal fullness, flatulence, abdominal bloating, bloody stools, fever
Shimizu et al., 2019 [42]	0	0	NR	NR

**Table 4:** Efficacy of FMT in pediatric IBD patients.

	Total Patients			Clinical Response			Clinical Remission			Both Response and Remission		
	**CD**	**UC**	**All**	**CD**	**UC**	**All**	**CD**	**UC**	**All**	**CD**	**UC**	**All**
Goyal et al., 2018 [38]	3	3	6	1	2	3	3	1	4	1	1	2
Karolewska et al., 2018 [37]	2	8	10	2	6	8	2	5	7	2	3	5
Suskind et al., 2015 [40]	9	0	9	4	0	4	7	0	7	4	0	4
Kunde et al., 2013 [41]	0	9	9	0	5	5	0	4	4	0	4	4
Total	14	20	34	7	13	20	12	10	22	7	8	15

**Table 5:** Outputs of sensitivity analysis (n = 11).

Studies	Pooled ES	LCI 95%	UCI 95%	Cochran Q	*p*	I^2^	I^2^ LCI 95%	I^2^ UCI 95%
Nicholson et al., 2022 [32]	0.317	0.117	0.517	93.065	0.000	90.329	84.357	94.022
Karolewska-Bochenek et al., 2021 [36]	0.269	0.125	0.412	86.671	0.000	89.616	83.035	93.644
Nicholson et al., 2020 [33]	0.329	0.145	0.513	71.939	0.000	87.489	79.009	92.544
Cho et al., 2019 [34]	0.311	0.160	0.462	94.497	0.000	90.476	84.626	94.100
Shimizu et al., 2019 [42]	0.328	0.175	0.481	91.947	0.000	90.212	84.140	93.959
Goyal et al., 2018 [38]	0.265	0.123	0.406	80.072	0.000	88.760	81.429	93.197
Karolewska-Bochenek et al., 2018 [37]	0.295	0.146	0.444	93.488	0.000	90.373	84.437	94.045
Fareed et al., 2018 [35]	0.323	0.173	0.473	93.259	0.000	90.349	84.394	94.032
Hourigan et al., 2015 [39]	0.323	0.173	0.473	93.259	0.000	90.349	84.394	94.032
Suskind et al., 2015	0.263	0.122	0.405	83.863	0.000	89.268	82.385	93.462
Kunde et al., 2013 [41]	0.221	0.105	0.336	49.061	0.000	81.656	67.406	89.675

## Data Availability

Data supporting the statements can be found on PubMed, EMBASE, CINAHL, Web of Science, Cochrane, and Google Scholar.

## References

[1] Chang J.T. (2020). Pathophysiology of Inflammatory Bowel Diseases. N. Engl. J. Med..

[2] Rosen M.J., Dhawan A., Saeed S.A. (2015). Inflammatory Bowel Disease in Children and Adolescents. JAMA Pediatr..

[3] Hazel K., O’Connor A. (2020). Emerging treatments for inflammatory bowel disease. Ther. Adv. Chronic Dis..

[4] Gupta S., Allen-Vercoe E., Petrof E.O. (2016). Fecal microbiota transplantation: In perspective. Ther. Adv. Gastroenterol..

[5] Thursby E., Juge N. (2017). Introduction to the human gut microbiota. Biochem. J..

[6] Rigottier-Gois L. (2013). Dysbiosis in inflammatory bowel diseases: The oxygen hypothesis. ISME J..

[7] Paramsothy S., Paramsothy R., Rubin D.T., Kamm M.A., Kaakoush N.O., Mitchell H.M., Castaño-Rodríguez N. (2017). Faecal Microbiota Transplantation for Inflammatory Bowel Disease: A Systematic Review and Meta-analysis. J. Crohns Colitis.

[8] Imdad A., Nicholson M.R., Tanner-Smith E.E., Zackular J.P., Gomez-Duarte O.G., Beaulieu D.B., Acra S. (2018). Fecal transplantation for treatment of inflammatory bowel disease. Cochrane Database Syst. Rev..

[9] Shi Y., Dong Y., Huang W., Zhu D., Mao H., Su P. (2016). Fecal Microbiota Transplantation for Ulcerative Colitis: A Systematic Review and Meta-Analysis. PLoS ONE.

[10] Derrien M., Alvarez A.S., de Vos W.M. (2019). The Gut Microbiota in the First Decade of Life. Trends Microbiol..

[11] Chen C.C., Chiu C.H. (2022). Current and future applications of fecal microbiota transplantation for children. Biomed. J..

[12] Tariq R., Syed T., Yadav D., Prokop L.J.M., Singh S.M., Loftus E.V.J., Pardi D.S.M., Khanna S.M. (2023). Outcomes of Fecal Microbiota Transplantation for C. difficile Infection in Inflammatory Bowel Disease: A Systematic Review and Meta-analysis. J. Clin. Gastroenterol..

[13] Wang A.Y., Popov J., Pai N. (2016). Fecal microbial transplant for the treatment of pediatric inflammatory bowel disease. World J. Gastroenterol..

[14] Hardin A.P., Hackell J.M., Committee on Practice and Ambulatory Medicine (2017). Age Limit of Pediatrics. Pediatrics.

[15] Abu-Zidan F.M., Abbas A.K., Hefny A.F. (2012). Clinical “case series”: A concept analysis. Afr. Health Sci..

[16] Sterne J.A.C., Savović J., Page M.J., Elbers R.G., Blencowe N.S., Boutron I., Cates C.J., Cheng H.Y., Corbett M.S., Eldridge S.M. (2019). RoB 2: A revised tool for assessing risk of bias in randomised trials. BMJ.

[17] Wells G., Shea B., O’Connell D., Peterson J., Welch V., Losos M., Tugwell P. (2013). The Newcastle-Ottawa Scale (NOS) for Assessing the Quality of Nonrandomised Studies in Meta-Analyses. http://www.ohri.ca/programs/clinical_epidemiology/oxford.asp.

[18] American College of Cardiology/American Heart Association Task Force on Practice Guidelines, Obesity Expert Panel, 2013 (2014). Expert Panel Report: Guidelines (2013) for the management of overweight and obesity in adults. Obesity.

[19] Turner D., Otley A.R., Mack D., Hyams J., de Bruijne J., Uusoue K., Walters T.D., Zachos M., Mamula P., Beaton D.E. (2007). Development, validation, and evaluation of a pediatric ulcerative colitis activity index: A prospective multicenter study. Gastroenterology.

[20] Hyams J.S., Ferry G.D., Mandel F.S., Gryboski J.D., Kibort P.M., Kirschner B., Griffiths A.M., Katz A.J., Grand R.J., Boyle J.T. (1991). Development and validation of a pediatric Crohn’s disease activity index. J. Pediatr. Gastroenterol. Nutr..

[21] Turner D., Griffiths A.M., Walters T.D., Seah T., Markowitz J., Pfefferkorn M., Keljo D., Otley A., Leleiko N., Mack D. (2010). Appraisal of the pediatric Crohn’s disease activity index on four prospectively collected datasets: Recommended cutoff values and clinimetric properties. Am. J. Gastroenterol..

[22] Kundhal P.S., Critch J.N., Zachos M., Otley A.R., Stephens D., Griffiths A.M. (2003). Pediatric Crohn Disease Activity Index: Responsive to short-term change. J. Pediatr. Gastroenterol. Nutr..

[23] DerSimonian R., Laird N. (1986). Meta-analysis in clinical trials. Control. Clin. Trials.

[24] Higgins J.P., Green S. (2011). Cochrane Handbook for Systematic Reviews of Interventions.

[25] Higgins J.P., Thompson S.G., Deeks J.J., Altman D.G. (2003). Measuring inconsistency in meta-analyses. BMJ.

[26] Kanwal F., White D. (2012). “Systematic Reviews and Meta-analyses” in Clinical Gastroenterology and Hepatology. Clin. Gastroenterol. Hepatol..

[27] Guyatt G.H., Oxman A.D., Kunz R., Woodcock J., Brozek J., Helfand M., Alonso-Coello P., Glasziou P., Jaeschke R., Akl E.A. (2011). GRADE guidelines: 7. Rating the quality of evidence--inconsistency. J. Clin. Epidemiol..

[28] Furuya-Kanamori L., Barendregt J.J., Doi S.A.R. (2018). A new improved graphical and quantitative method for detecting bias in meta-analysis. Int. J. Evid. Based Healthc..

[29] Sterne J.A., Egger M. (2001). Funnel plots for detecting bias in meta-analysis: Guidelines on choice of axis. J. Clin. Epidemiol..

[30] Fagan T. (1999). Exact 95% confidence intervals for differences in binomial proportions. Comput. Biol. Med..

[31] Lin L. (2019). Use of Prediction Intervals in Network Meta-analysis. JAMA Netw. Open.

[32] Nicholson M.R., Alexander E., Ballal S., Davidovics Z., Docktor M., Dole M., Gisser J.M., Goyal A., Hourigan S.K., Jensen M.K. (2022). Efficacy and Outcomes of Faecal Microbiota Transplantation for Recurrent Clostridioides difficile Infection in Children with Inflammatory Bowel Disease. J. Crohns Colitis.

[33] Nicholson M.R., Mitchell P.D., Alexander E., Ballal S., Bartlett M., Becker P., Davidovics Z., Docktor M., Dole M., Felix G. (2020). Efficacy of Fecal Microbiota Transplantation for Clostridium difficile Infection in Children. Clin. Gastroenterol. Hepatol..

[34] Cho S., Spencer E., Hirten R., Grinspan A., Dubinsky M.C. (2019). Fecal Microbiota Transplant for Recurrent Clostridium difficile Infection in Pediatric Inflammatory Bowel Disease. J. Pediatr. Gastroenterol. Nutr..

[35] Fareed S., Sarode N., Stewart F.J., Malik A., Laghaie E., Khizer S., Yan F., Pratte Z., Lewis J., Immergluck L.C. (2018). Applying fecal microbiota transplantation (FMT) to treat recurrent Clostridium difficile infections (rCDI) in children. PeerJ.

[36] Karolewska-Bochenek K., Lazowska-Przeorek I., Grzesiowski P., Dziekiewicz M., Dembinski L., Albrecht P., Radzikowski A., Banaszkiewicz A. (2021). Faecal Microbiota Transfer—A new concept for treating cytomegalovirus colitis in children with ulcer-ative colitis. Ann. Agric. Environ. Med..

[37] Karolewska-Bochenek K., Grzesiowski P., Banaszkiewicz A., Gawronska A., Kotowska M., Dziekiewicz M., Albrecht P., Radzikowski A., Lazowska-Przeorek I. (2018). A Two-Week Fecal Microbiota Transplantation Course in Pediatric Patients with In-flammatory Bowel Disease. Adv. Exp. Med. Biol..

[38] Goyal A., Yeh A., Bush B.R., Firek B., Siebold L.M., Rogers M.B., Kufen R.B.A.D., Morowitz M.J. (2018). Safety, Clinical Response, and Microbiome Findings Following Fecal Microbiota Transplant in Children with Inflammatory Bowel Disease. Inflamm. Bowel Dis..

[39] Hourigan S.K., Chen L.A., Grigoryan Z., Laroche G., Weidner M., Sears C.L., Oliva-Hemker M. (2015). Microbiome changes associated with sustained eradication of Clostridium difficile after single faecal microbiota transplantation in children with and without inflammatory bowel disease. Aliment. Pharmacol. Ther..

[40] Suskind D.L., Brittnacher M.J., Wahbeh G., Shaffer M.L., Hayden H.S., Qin X., Singh N., Damman C.J., Hager K.R., Nielson H. (2015). Fecal microbial transplant effect on clinical outcomes and fecal microbiome in active Crohn’s disease. Inflamm. Bowel Dis..

[41] Kunde S., Pham A., Bonczyk S., Crumb T., Duba M., Conrad H., Cloney D., Kugathasan S. (2013). Safety, tolerability, and clinical response after fecal transplantation in children and young adults with ulcerative colitis. J. Pediatr. Gastroenterol. Nutr..

[42] Shimizu H., Ohnishi E., Arai K., Takeuchi I., Kamura H., Hata K. (2019). P043 Outcome of the repetitive fecal microbiota trans-plantation using fecal solution prepared under the anaerobic condition following the antibiotic pretreatment in eight children with ulcerative colitis. Inflamm. Bowel Dis..

[43] Chauhan U., Popov J., Farbod Y., Kalantar M., Wolfe M., Moayyedi P., Marshall J.K., Halder S., Kaasalainen S. (2021). Fecal Microbiota Transplantation for the Treatment of Ulcerative Colitis: A Qualitative Assessment of Patient Perceptions and Ex-periences. J. Can. Assoc. Gastroenterol..

[44] Popov J., Hartung E., Hill L., Chauhan U., Pai N. (2021). Pediatric Patient and Parent Perceptions of Fecal Microbiota Transplan-tation for the Treatment of Ulcerative Colitis. J. Pediatr. Gastroenterol. Nutr..

[45] Wei Z.J., Dong H.B., Ren Y.T., Jiang B. (2022). Efficacy and safety of fecal microbiota transplantation for the induction of remission in active ulcerative colitis: A systematic review and meta-analysis of randomized controlled trials. Ann. Transl. Med..

[46] Food and Drug Administration Center for Biologics Evaluation Research Information on Additional Safety Protections for Use of F.M.T.U.S.. https://www.fda.gov/vaccines-blood-biologics/safety-availability-biologics/information-pertaining-additional-safety-protections-regarding-use-fecal-microbiota-transplantation.

[47] Tan X.Y., Xie Y.J., Liu X.L., Li X.Y., Jia B. (2022). A Systematic Review and Meta-Analysis of Randomized Controlled Trials of Fecal Microbiota Transplantation for the Treatment of Inflammatory Bowel Disease. Evid Based Complement. Alternat. Med..

[48] Wopereis H., Oozeer R., Knipping K., Belzer C., Knol J. (2014). The first thousand days—Intestinal microbiology of early life: Es-tablishing a symbiosis. Pediatr. Allergy Immunol..

[49] Thriene K., Michels K.B. (2023). Human Gut Microbiota Plasticity throughout the Life Course. Int. J. Environ. Res. Public Health.

[50] Mohammadkhah A.I., Simpson E.B., Patterson S.G., Ferguson J.F. (2018). Development of the Gut Microbiome in Children, and Lifetime Implications for Obesity and Cardiometabolic Disease. Children.

[51] Okahara K., Ishikawa D., Nomura K., Ito S., Haga K., Takahashi M., Shibuya T., Osada T., Nagahara A. (2020). Matching between Donors and Ulcerative Colitis Patients Is Important for Long-Term Maintenance after Fecal Microbiota Transplantation. J. Clin. Med..

[52] Pickard J.M., Zeng M.Y., Caruso R., Núñez G. (2017). Gut microbiota: Role in pathogen colonization, immune responses, and inflammatory disease. Immunol. Rev..

[53] Hansen R., Russell R.K., Reiff C., Louis P., McIntosh F., Berry S.H., Mukhopadhya I., Bisset M.W., Barclay A.R., Bishop J. (2012). Microbiota of de-novo pediatric IBD: Increased Faecalibacterium prausnitzii and reduced bacterial diversity in Crohn’s but not in ulcerative colitis. Am. J. Gastroenterol..

[54] Popov J., Caputi V., Nandeesha N., Rodriguez D.A., Pai N. (2021). Microbiota-Immune Interactions in Ulcerative Colitis and Colitis Associated Cancer and Emerging Microbiota-Based Therapies. Int. J. Mol. Sci..

[55] Li Q., Ding X., Liu K., Marcella C., Liu X., Zhang T., Liu Y., Li P., Xiang L., Cui B. (2020). Fecal Microbiota Transplantation for Ulcerative Colitis: The Optimum Timing and Gut Microbiota as Predictors for Long-Term Clinical Outcomes. Clin. Transl. Gastroenterol..

[56] Gevers D., Kugathasan S., Denson L.A., Vázquez-Baeza Y., Van Treuren W., Ren B., Schwager E., Knights D., Song S.J., Yassour M. (2014). The treatment-naive microbiome in new-onset Crohn’s disease. Cell Host Microbe.

[57] Nishihara Y., Ogino H., Tanaka M., Ihara E., Fukaura K., Nishioka K., Chinen T., Tanaka Y., Nakayama J., Kang D. (2021). Mucosa-associated gut microbiota reflects clinical course of ulcerative colitis. Sci. Rep..

[58] Paramsothy S., Kamm M.A., Kaakoush N.O., Walsh A.J., van den Bogaerde J., Samuel D., Borody T.J. (2017). Multidonor intensive faecal microbiota transplantation for active ulcerative colitis: A randomised placebo-controlled trial. Lancet.

[59] Shaw K.A., Bertha M., Hofmekler T., Chopra P., Vatanen T., Srivatsa A. (2016). Dysbiosis, inflammation, and response to treat-ment: A longitudinal study of pediatric subjects with newly diagnosed inflammatory bowel disease. Genome Med..

[60] Pai N., Popov J., Hill L., Hartung E., Grzywacz K., Moayyedi P., Surette M., Lee C., Godin D., Szamosi J. (2021). Results of the First Pilot Randomized Controlled Trial of Fecal Microbiota Transplant in Pediatric Ulcerative Colitis: Lessons, Limitations, and Future Prospects. Gastroenterology.

[61] Gill M., Blacketer C., Chitti F., Telfer K., Papanicolas L., Dann L.M., Tucker E.C., Bryant R.V., Costello S.P. (2020). Physician and patient perceptions of fecal microbiota transplant for recurrent or refractory Clostridioides difficile in the first 6 years of a central stool bank. JGH Open.

[62] Liu Y., Alnababtah K., Cook S., Yu Y. (2021). Healthcare providers’ perception of faecal microbiota transplantation with clostridium difficile infection and inflammatory bowel disease: A quantitative systematic review. Ther. Adv. Gastroenterol..

[63] Gundling F., Roggenbrod S., Schleifer S., Sohn M., Schepp W. (2018). Patient perception and approval of faecal microbiota trans-plantation (FMT) as an alternative treatment option for obesity. Obes. Sci. Pract..

